# Case report: Magnetic resonance imaging features with postoperative improvement of atypical cervical glioma characterized by predominant extramedullary distribution in a dog

**DOI:** 10.3389/fvets.2024.1400139

**Published:** 2024-05-22

**Authors:** Junyoung Kim, Kihoon Kim, Dai Jung Chung, Yebeen Kim, Kitae Kim, Dayoung Oh, Namsoon Lee, Jihye Choi, Junghee Yoon

**Affiliations:** ^1^N Animal Medical Center, Seoul, Republic of Korea; ^2^College of Veterinary Medicine and the Research Institute for Veterinary Science, Seoul National University, Seoul, Republic of Korea; ^3^Jeil Referral Animal Medical Center, Busan, Republic of Korea; ^4^Veterinary Health Center, University of Missouri, Colombia, MO, United States; ^5^Department of Environmental and Radiological Health Sciences, College of Veterinary Medicine and Bio-medical Sciences, Colorado State University, Fort Collins, CO, United States; ^6^College of Veterinary Medicine, Chungbuk National University, Cheongju, Republic of Korea

**Keywords:** canine, histopathology, laminotomy, oligodendroglioma, spinal tumors

## Abstract

**Introduction:**

Intramedullary cord tumors present diagnostic and therapeutic challenges. Furthermore, spinal cord tumors can move across compartments, making antemortem diagnosis difficult, even with advanced imaging. This report presents a rare case of a cranial cervical spinal glioma, confirmed by surgical histopathology, with postoperative improvement in a dog.

**Case description:**

A 9-year-old female Maltese dog presented with kyphotic posture, progressive left hemiparesis, and decreased appetite. Neurological examination revealed neck pain and decreased proprioception in the left limbs along with intact deep pain perception. Two days later, the patient developed non-ambulatory tetraparesis. Magnetic resonance imaging (MRI) revealed an ovoid, well-defined mass with homogeneously marked contrast enhancement in the second cervical spinal cord that severely compressed the spinal cord. This mass was heterogeneously hyperintense on T2-weighted images and iso-to-hypointense on T1-weighted images, showing an appearance resembling the “golf-tee” and “dural tail” signs. The MRI findings suggested an intradural extramedullary tumor. Intraoperatively, a well-demarcated mass which was locally adherent to the spinal meninges was removed. Both histopathological and genomic tumor tests were indicative of a glioma. Approximately 2 weeks postoperatively, the patient’s neurological signs returned to normal.

**Conclusion:**

This case report describes an atypical cervical glioma with complicated MR characteristics in a dog, where MRI helped guide surgical intervention.

## 1 Introduction

Spinal neoplasia can result in clinical signs of spinal cord dysfunction (1). Spinal cord tumors are classified as intramedullary (accounting for ~15% of such tumors) (1–8), extradural (~50%) (1, 4, 5, 8), and intradural extramedullary tumors (35%). Intramedullary tumors include astrocytomas, oligodendrogliomas, and ependymomas (1, 5–7). Some neoplasms, including nephroblastomas and peripheral nerve sheath tumors, can occupy both intradural extramedullary and intramedullary locations (2, 5, 6).

Magnetic resonance imaging (MRI) has excellent contrast resolution and is essential for the diagnosis of spinal cord neoplasms (6, 9). Signal intensity, degree of contrast enhancement, and presence of fluid-filled compartments are used to differentiate spinal cord neoplasms (5). Moreover, the location of a spinal cord neoplasm with respect to the meninges helps predict the histological type (1, 5). However, intramedullary cord tumors present diagnostic and therapeutic challenges (9). Spinal cord tumors can move across compartments, making antemortem diagnosis difficult, even with advanced imaging (8). Although successful surgical removal has been described, intramedullary spinal cord tumors may not be generally amenable to surgery (1, 5, 8).

Treatment options for spinal tumors include surgical removal, radiotherapy, and chemotherapy (1). The prognosis depends on the tumor type, degree of spinal infiltration, spinal cord damage pre-and intraoperatively, degree of local resection, and surgeon’s experience (1, 2, 5). Cytoreductive surgery, the primary treatment for patients with spinal cord neoplasia, may be used with or without radio-and chemotherapy (1, 5). It is commonly performed for extradural and intradural extramedullary neoplasms but rarely for intramedullary neoplasms (1, 2, 5). This reflects the technical expertise required to resect neoplasms within the spinal cord without iatrogenic injuries. Therefore, the preoperative distinction between intradural extramedullary and intramedullary neoplasms may be useful. Although MRI has excellent contrast resolution, its relatively low spatial resolution may negatively affect differentiation between intradural extramedullary and intramedullary neoplasms (5). A previous canine study reported a case of confirmed intramedullary cervical glioma exhibiting characteristics of intradural extramedullary lesions on MRI. In humans, MRI has a sensitivity of ~83% for the diagnosis of intradural extramedullary tumors, with 31 of 187 of these tumors misdiagnosed as intramedullary tumors (5). The present report describes a rare case of postoperative improvement in a cranial cervical glioma in a dog characterized by a predominant intradural extramedullary lesion distribution on MRI findings regarding the origin of the mass.

## 2 Case description

A 9-year-old intact female Maltese dog weighing 1.9 kg was presented with a kyphotic posture and decreased appetite for 3 days. Neurological examination revealed no abnormalities other than mild neck pain. A nonsteroidal anti-inflammatory drug was administered for pain control. However, 2 days later, the patient was returned to the hospital with deteriorating neurological symptoms and left hemiparesis. The dog was ambulatory with mild-to-moderate neck pain and decreased proprioceptive reactions in the left limbs, along with intact deep pain perception. Physical examination revealed a grade II heart murmur with no other abnormalities. Laboratory findings were mostly normal, except for mildly elevated levels of alkaline phosphatase (405 U/L; reference range, 47–254 U/L) and alanine aminotransferase (95 U/L; reference range, 17–78 U/L). Radiography revealed marked ventral spondylosis deformans with intervertebral disc space narrowing in the 6th–7th cervical vertebrae, with no other abnormalities. Echocardiography and abdominal ultrasonography showed myxomatous mitral valve disease stage B1 and small bilateral renal calculi. Based on clinical and neurological signs, the main differential diagnoses were cervical intervertebral disc disease, degenerative myelopathy, neoplasia or inflammatory diseases, and vascular disorders of the brain or cervical spine. Therefore, an MRI of the brain and cervical spinal cord was planned. Two days before the MRI examination, the patient’s clinical signs progressively deteriorated, presenting as non-ambulatory tetraparesis with normal deep pain responses in all four limbs.

**Figure 1:**
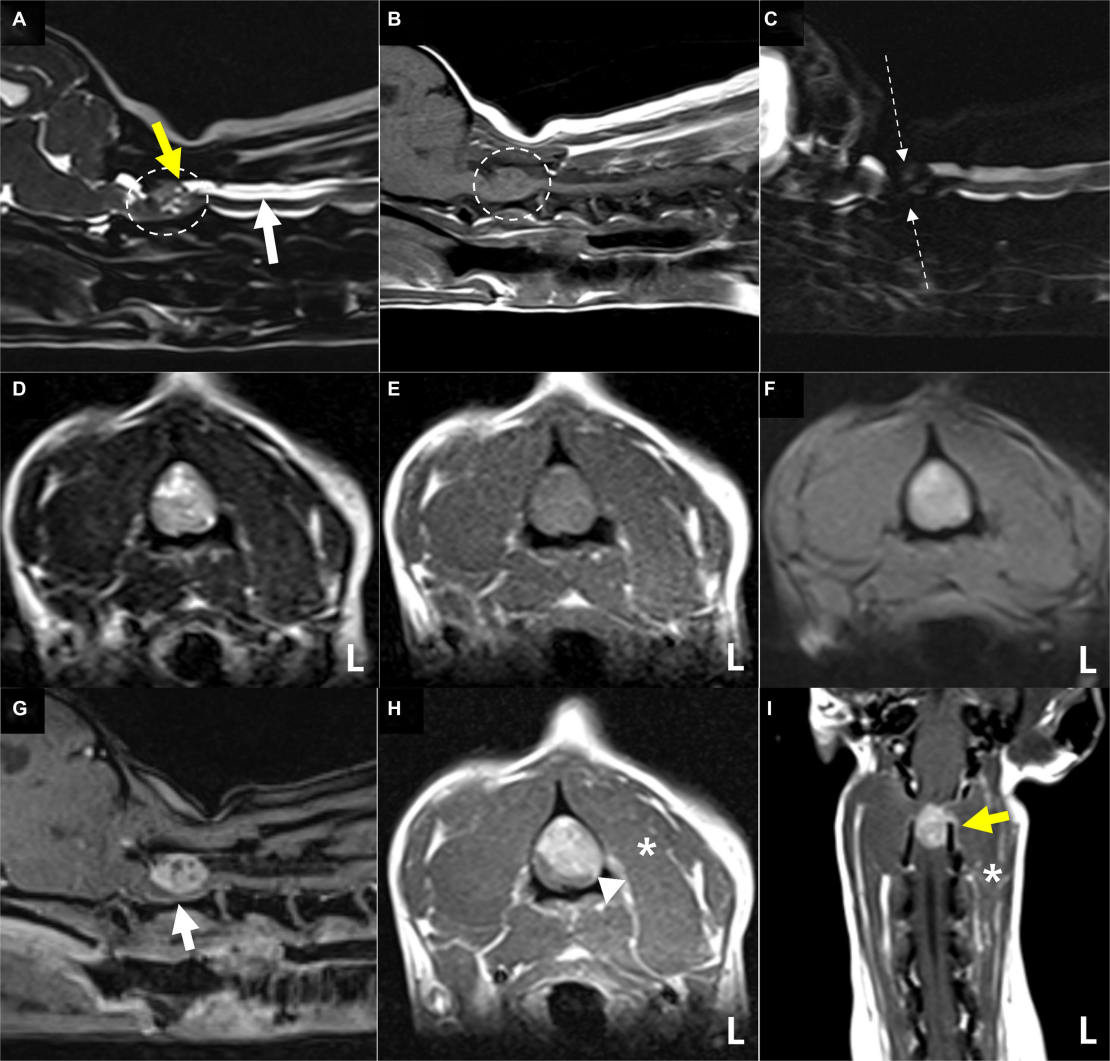
Magnetic resonance images showing the cervical spinal cord of the present case. The extensive lesion in the second cervical (C2) spinal cord **(A,B)** (dotted circles) is ill-defined and heterogeneously hyperintense with loss of normal intraparenchymal architecture on sagittal **(A)** and transverse **(D)** T2-weighted images. The lesion appears iso-to-hypointense on sagittal **(B)** and transverse **(E)** T1-weighted images and iso-to-hyperintense on T2-weighted multi-echo data image combination sequence images **(F)**. The origin of this C2 lesion cannot be determined due to its extensive distribution throughout the spinal parenchyma on all images. The lesion leads to focal spinal cord swelling and attenuation of the cerebrospinal fluid line on T2-weighted half-Fourier acquisition single-shot turbo spin-echo myelography **(C)**, accompanied by the presence of a structure resembling the “golf-tee” sign **(A)** (yellow arrow) in the dorsal subarachnoid space and diffuse syringomyelia **(A)** (white arrow) just caudal to the lesion. Post-contrast T1-weighted sagittal **(G)**, transverse **(H)**, and dorsal **(I)** images of the cervical spinal cord. Post-contrast T1-weighted images reveal an ovoid, well-defined mass with homogeneously marked contrast enhancement. The mass contains multiple small cavities **(G)**, suggestive of cystic or necrotic areas, leading to severe left-sided compression of the spinal cord and an appearance mimicking the “dural tail sign” of the adjacent meninges **(H)** (arrowhead) **(H)**. A thickened left C2 nerve root with contrast enhancement (**I**) (yellow arrow), mild atrophy and contrast enhancement of the paraspinal muscle **(H,I)** (asterisks) adjacent to the lesion, and ventral meningeal enhancement **(G)** (white arrow) are also present. These findings strongly suggest a spinal tumor concurrent with gliosis and left-sided neuritis and myositis rather than inflammatory or vascular diseases. Based on these MRI findings, the mass is considered to be of intradural extramedullary rather than of intramedullary origin.

**Figure 2:**
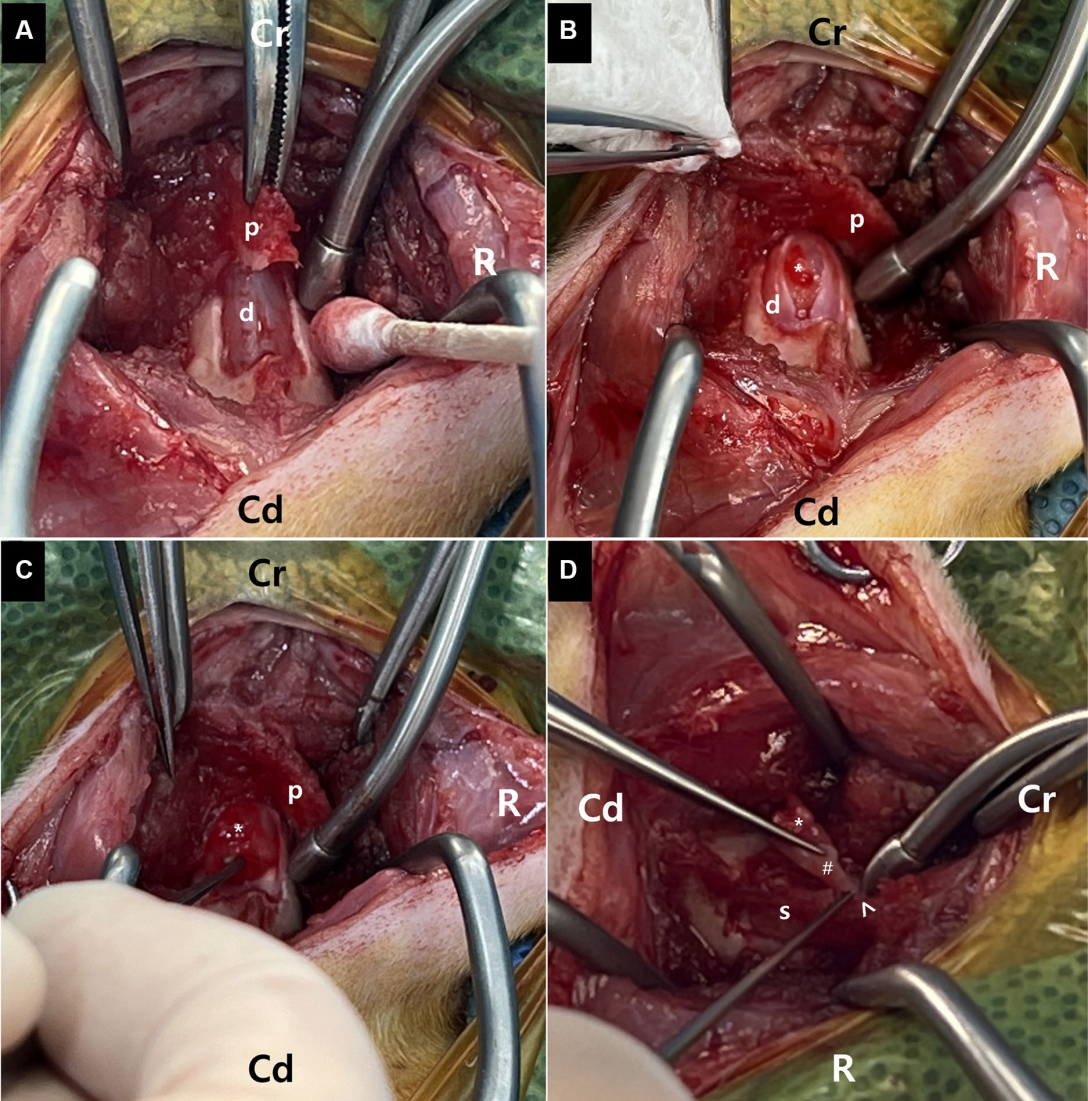
Surgical removal of the mass following dorsal laminotomy of the axis combined with durotomy at the second cervical vertebra. **(A)** A bone flap (p) of the C2 axis spinous process is created with laminotomy of the C2 axis. The bone flap (p) is rotated dorsally and cranially to visualize the dorsal dura **(D)**. **(B,C)** Following the laminotomy, a midline durotomy reveals a capsulated mass (asterisk). Most parts of this tumor are easily removed from the adjacent spinal cord (s) parenchyma. **(D)** The ventral part of the mass (asterisk) regionally adheres to the spinal cord (S) meninges (arrowhead) adjacent to the left nerve root with fibrotic tissues (hash). The ventral tumor tissues are carefully removed.

**Figure 3:**
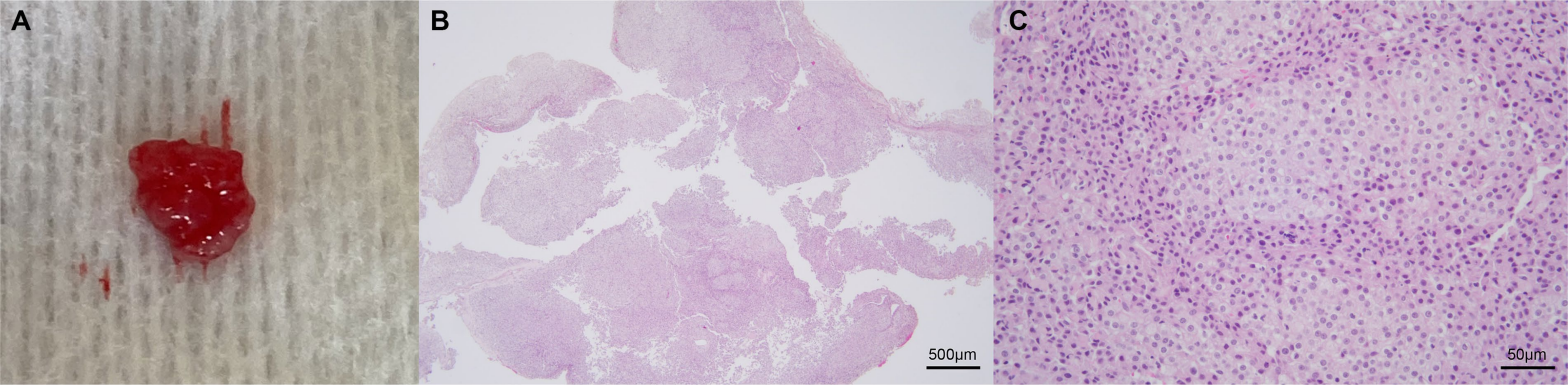
Representative images of the excised mass. Gross appearance **(A)** and photomicrographs of histopathological evaluation **(B,C)**. The mass is characterized by a red, well-circumscribed, and elastic appearance **(A)**. The poorly demarcated mass is composed of neoplastic cells arranged in sheets and occasional short streams (**B**: 40×). The spindled, round, or polygonal cells contain small-to-moderate amounts of pale eosinophilic, wispy-to-granular cytoplasm, and heterochromatic nuclei with one or two variably distinct nucleoli (**C**: 400×). Anisocytosis and anisokaryosis are moderate **(C)**. These findings are consistent with glioma, in which oligodendroglioma may be the most consistent. Scale bars in **(B)**: 500 μm and in **(C)**: 50 μm.

MR images of the brain and cervical spine were acquired using a 1.5-Tesla scanner (MAGNETOM AVANTO, Siemens, Germany) with head and neck knee coils, respectively. The MRI sequences of the brain and cervical spine used in the present case are shown in Table 1. On T2-weighted images, ill-defined, heterogeneously hyperintense areas in the spinal cord were identified eccentrically in the central-to-left dorsal aspect of the second cervical (C2) spinal cord, leading to focal spinal cord swelling showing a loss of normal parenchymal architecture and circumferential attenuation of the cerebrospinal fluid (CSF) line on T2-weighted half-Fourier acquisition single-shot turbo spin-echo sequence (Figures 1A,C,D). The lesion appeared iso-to-hypointense on T1-weighted images and iso-to-hyperintense on T2-weighted multi-echo data image combination sequence images (Figures 1B,E,F). Post-contrast T1-weighted images revealed an ovoid, well-defined mass with homogeneously marked contrast enhancement (size: 10.9 × 7.2 × 8.3 mm, length/height/width; Figures 1G–I). The mass contained multiple small cavitary lesions suggestive of cystic or necrotic areas, causing severe left-sided compression of the spinal cord (Figure 1G). In the dorsal subarachnoid space just caudal to the C2 mass, although not typical, a structure resembling the “golf-tee” sign, a characteristic sign of an intradural extramedullary lesion (4, 6, 10, 11), and syringomyelia were noted (Figure 1A). Additionally, an appearance mimicking the “dural tail sign” in the meninges adjacent to the thickened C2 left nerve root with contrast enhancement, mild atrophy with contrast enhancement of the paraspinal muscle adjacent to the lesion, and ventral meningeal enhancement were identified (Figures 1H,I). However, broad-based dural attachment of the mass was not evident in any sequence. Other MRI findings included multiple cervical intervertebral disc degeneration with protrusion, bilateral ventriculomegaly, and Chiari-like malformations. Based on MRI findings, we concluded that the C2 spinal mass was the cause of the patient’s symptoms. Considering the MRI characteristics of the mass (single, well-defined, marked contrast enhancement, and mass effect), a spinal tumor concurrent with gliosis, left-sided neuritis, and myositis was strongly suspected rather than inflammatory or vascular diseases. However, the origin of the C2 mass could not be determined because of its extensive distribution throughout the spinal parenchyma in all images. An intradural extramedullary tumor (e.g., meningioma, nerve sheath tumor, or nephroblastoma) was considered a primary differential diagnosis, and other possibilities were an intramedullary tumor or intramedullary invasion by an extramedullary tumor. Given its clear margins, location, size, and the progression of clinical signs, surgical removal of the mass was planned via dorsal laminectomy of C2 combined with durotomy. A high-speed burr and bone-cutting forceps were used to create a hinged osteotomy of the C2 dorsal arch involving the cranial 75% of the C2 spinous process (Figure 2). The bone flap was rotated dorsally and cranially on the preserved attachment of the cranial aspect of the C2 spinous process to the dorsal arch of C1 to visualize the dorsal dura. A midline durotomy revealed a capsulated mass. Most tumor parts were easily separated and removed from the adjacent spinal cord parenchyma; however, the ventral part adhered locally to the spinal cord meninges adjacent to the left nerve root with fibrotic tissues (Figure 2). After careful removal of these ventral tumor tissues, mild hemorrhage occurred but was promptly controlled. No abnormalities were observed in the spinal cord. A biological, absorbable dura substitute (Lyoplant Onlay; B Braun Co., Germany) was used to cover the dural defect and sutured to the adjacent dura before the spinous process was returned to its normal position. Subsequently, the dorsal arch of the axis was rotated back over the exposed vertebral canal, and the spinous process was stabilized with 2–0 non-absorbable sutures (PROLENE Polypropylene Suture; ETHICON Co., United States) through one predrilled hole. The excised mass, characterized by a red, well-circumscribed, and elastic appearance (Figure 3), was histopathologically examined (hematoxylin and eosin staining). Spindle round-to-polygonal cells containing small-to-moderate amounts of pale, eosinophilic, wispy-to-granular cytoplasm were observed. The nuclei were heterochromatic, with one or two variably distinct nucleoli, along with moderate anisocytosis and anisokaryosis (Figure 3). These findings were most consistent with glioma, in which oligodendroglioma may be the most consistent. The histopathological identification of an intramedullary tumor did not match the MRI or surgical findings. Thus, a genomic tumor test was performed using the same tissue sample as the histopathology; immunohistochemical staining for glial cell tumor markers could not be performed because of the owner’s financial constraints. This genomic tumor test is a tumor-only, next-generation sequencing, hybrid-capture test involving canine gene panel covering 482,000 base pairs of 120 genes associated with canine or human cancer. Genetic data revealed copy number loss of *CDKN2N* and gain of *MYC*, supporting the diagnosis of a canine glioma. Three days postoperatively, the dog was able to stand up and walk in the cage, and its appetite returned to normal. Approximately 2 weeks postoperatively, all neurological signs returned to normal. Although postoperative MRI and computed tomography (CT) examinations were recommended to confirm the residual tissue of the glioma and assess metastasis, including “CSF drop metastasis” from the glioma (12–14), only a 16-channel multi-detector CT (Emotion 16, Siemens Healthcare, Forchheim, Germany) scan was performed due to the owner’s financial constraints. On postoperative CT images, regional bone defects in the C2 lamina due to surgery were identified. However, no significant CT findings were identified in the whole body, including tumor metastasis, lymph node enlargement, or contrast-enhanced lesions around the surgical site. Although chemotherapy was recommended based on the genetic data and considering possible recurrence and local adhesion or invasion of the tumor, the owner rejected this suggestion. Ten months after discharge, no recurrence of neurological signs had been reported.

**Table 1:** MRI sequences of the brain and cervical spine used in the present case.

	Sequences	TR (ms)	TE (ms)	FOV (mm)	Slice thickness (mm)
Brain	T1 sagittal	560	12	130 × 130	3.0
	T2 sagittal	3,860	120	130 × 130	3.0
	T1 transverse	599	11	130 × 130	3.0
	T2 transverse	4,000	89	130 × 130	3.0
	FLAIR transverse	7,290	88	130 × 130	3.0
	T2* GE transverse	572	15	130 × 130	3.0
	DWI transverse	4,500	91	130 × 130	3.0
Cervical	T1 sagittal	524	13	160 × 160	2.5
	T2 sagittal	3,000	91	160 × 160	2.5
	T1 transverse	550	15	100 × 100	3.0
	T2 transverse	3,360	82	100 × 100	3.0
	T2 MEDIC transverse	600	23	120 × 120	3.0
	3D T2 SPACE sagittal	1730	193	160 × 160	0.9
	T2 HASTE myelography sagittal	8,000	1,200	160 × 160	
Post-contrast	T1 sagittal	524	13	160 × 160	2.5
	T1 transverse	550	15	100 × 100	3.0
	T1 dorsal	400	12	180 × 180	3.0
	3D T1^a^ sagittal	21	7	180 × 180	0.8

DWI, diffusion-weighted images; FLAIR, fluid-attenuated inversion recovery; FOV, field of view; HASTE, half-Fourier acquisition single-shot turbo spin-echo; MRI, magnetic resonance imaging; MEDIC, multi-echo data image combination; SPACE, sampling perfection with application-optimized contrast using different flip angle evolution; T2* GE, T2*-weighted gradient-recalled echo; TE, echo time; TR, repetition time; 3D T1^a^, three-dimensional T1-weighted spoiled gradient-echo water excitation.

The authors declare that no Institutional Animal Care and Use Committee or other approval was needed. Written informed consent was obtained from the owner prior to any procedures being performed.

## 3 Discussion

This report presents a rare case of a cranial cervical spinal glioma, confirmed by surgical histopathology, with postoperative improvement in a dog. Gliomas arise from glial cells in the brain or spinal cord and are grouped as astrocytic, oligodendroglial, and ependymal tumors. Canine gliomas typically affect older individuals, especially those of brachycephalic breeds, and occur mainly in the frontal, parietal, or temporal telencephalic lobes and less frequently in the brainstem and spinal cord (3, 7). The most common type of canine spinal cord glioma is ependymoma, followed by astrocytoma, oligodendroglial tumors, gliomatosis cerebri, and unclassified glioma (3, 8). In a recent report of seven canine spinal gliomas, the affected spinal cord segments were, in decreasing order of frequency, thoracic (three cases), lumbar (three cases), and cervical (one case); the most common spinal cord glioma was oligodendroglioma (3). In another canine report of 53 intramedullary spinal tumor cases, thoracolumbar segments were the most frequently affected, although primary intramedullary tumors were more common in the cervical spinal cord in young dogs. Furthermore, intramedullary masses were identified in all dogs in which MRI was performed (8). However, intramedullary spinal tumors can expand and infiltrate other sites, although intramedullary spinal lesions may initially be intraparenchymal (3, 8). Clinical diagnosis and treatment of intramedullary neoplasms may be challenging because of the rapidly progressive clinical course and neuroanatomical location of these tumors. On MRI, both astrocytomas and oligodendrogliomas appear as ovoid-to-elliptical mass lesions that are well-marginated, located eccentrically in the spinal cord, and associated with variable degrees of spinal cord expansion. Astrocytomas and oligodendrogliomas are iso-to-hypointense on T1-weighted images and hyperintense on T2-weighted and short-tau inversion recovery images with moderate contrast enhancement. In contrast, ependymomas appear as focal to multi-segmental, fusiform, centrally located lesions that are heterogeneously iso-to-hypointense on T1-weighted images and hyperintense on T2-weighted images, with marked contrast enhancement (8). Nevertheless, there have been reports of one dog diagnosed with oligodendroglioma showing hyperintensity on T1-weighted images and another dog with diffuse leptomeningeal oligodendroglioma accompanied by the “dural tail sign” (15, 16). In a recent study evaluating the MRI characteristics of inflammatory, neoplastic, and vascular diseases of the canine spinal cord, the diagnostic sensitivity was excellent for intradural spinal diseases but poor for intramedullary tumors. Published descriptions of the imaging features of intramedullary tumors are sparse, their predilection sites are poorly understood and variable, and their MRI features overlap with those of inflammatory and vascular diseases (9). Therefore, the present case, which included MRI and surgical resection with histopathological examination of the spinal mass, has significant clinical value. The MRI characteristics included a well-defined, homogenously marked contrast-enhancing, single-mass lesion with extensive, eccentric distribution throughout the spinal parenchyma. The origin of the lesion was difficult to establish because of the extensive involvement of the spinal cord parenchyma without broad-based dural contact and findings mimicking the “golf-tee” and “dural tail” signs, further complicating the evaluation. This case suggests that an intramedullary glioma should be considered when the lesion is widely distributed throughout the spinal parenchyma, even if it is located eccentrically rather than centrally. The authors considered the present case to be a glioma originating from the spinal cord parenchyma, exhibiting extensive expansion in the extramedullary space. Ultimately, the mass was relatively well removed, significantly contributing to the improvement of the neurological symptoms. Intraoperatively, the mass was observed in the extramedullary region, facilitating its identification and resection after durotomy. The left ventral area of the mass adhered to the meninges around the left intervertebral foramen. To date, such canine spinal gliomas attached to the meninges with a pedunculated pattern have not been reported. Additionally, the mass exhibited a well-circumscribed red-colored and elastic appearance, differing in characteristics from those in previously reported soft grayish masses in oligodendroglioma or oligodendrogliomatosis (14–16). Consequently, the surgeons suspected, even postoperatively, the extension of an intradural extramedullary tumor into the intramedullary parenchyma rather than an intramedullary origin. Considering the craniocervical location and elastic appearance of the mass, the surgeons suspected a meningioma (17, 18). Following the histopathological results, a genomic tumor test was performed, which strongly supported the diagnosis of a spinal glioma. Indeed, it has been reported that the MYC gene is overexpressed in glioma, and the deletion of the CDKN gene is considered a clinically important molecular alteration in glioma (19, 20). This case provided additional evidence against drawing a conclusion regarding a presumptive histologic diagnosis and making decisions based solely on MRI findings to classify the lesion location relative to the meninges (5).

To the best of our knowledge, this is the only reported case of a canine spinal glioma with remarkable postoperative improvement. Most reported canine spinal gliomas or intramedullary tumors were diagnosed postmortem or were associated with a short survival time due to rapid deterioration postoperatively (3, 8, 13–16, 21). This may be primarily due to the challenge of diagnosing spinal glioma antemortem and implementing surgical treatment owing to the intramedullary tumor location.

This report had several limitations, including a lack of immunohistochemical data, CSF analysis, and various MRI sequences. Although differentiating among glioma types based on histopathological results was necessary, immunohistochemistry could not be performed. Therefore, the definitive glioma type could not be confirmed. Nevertheless, we considered the mass to be an oligodendroglioma or astrocytoma with higher potential, based on MRI features and histopathological findings. Considering the size, distribution, and pronounced contrast enhancement, the mass leaned more toward a high-grade tumor than a low-grade tumor, indicating the necessity for ongoing monitoring to detect any potential recurrence (16). The lack of CSF analyses might not have substantially impacted the patient’s evaluation and diagnosis because spinal gliomas have no specific CSF characteristics (3, 7, 9, 14, 22). The inclusion of post-contrast fat saturation and diffusion tensor imaging may have provided additional information on the mass distribution and location and may have proven beneficial in determining the type of spinal tumors on MRI for future reference (23, 24).

Despite numerous reports detailing the MRI features of canine spinal tumors, the present case indicated the existence of additional types not yet reported. Although histopathological examination is typically required for a definitive diagnosis, MRI provided detailed information on the location, distribution, and margination of spinal cord lesions, facilitating surgical decision-making. Additional information regarding spinal tumor characteristics on MRI may contribute to the advancement of antemortem diagnosis. Furthermore, surgical treatment may benefit patients with spinal cord tumors that are considered removable based on MRI findings.

## Data availability statement

The original contributions presented in the study are included in the article/supplementary material, further inquiries can be directed to the corresponding author/s.

## Ethics statement

Ethics review and approval was not required as per local legislation and institutional requirements. Written informed consent was obtained from the owner prior to any procedures being performed. Written informed consent was obtained from the owners for the participation of their animals in this study.

## Author contributions

JK: Conceptualization, Data curation, Formal analysis, Investigation, Writing – original draft, Writing – review & editing. KihK: Conceptualization, Data curation, Formal analysis, Investigation, Writing – review & editing. DC: Data curation, Formal analysis, Investigation, Writing – review & editing. YK: Data curation, Formal analysis, Investigation, Writing – review & editing. KitK: Data curation, Formal analysis, Investigation, Writing – review & editing. DO: Data curation, Formal analysis, Investigation, Writing – review & editing. NL: Data curation, Formal analysis, Investigation, Writing – review & editing. JC: Data curation, Formal analysis, Investigation, Writing – review & editing. JY: Investigation, Resources, Supervision, Validation, Writing – review & editing.
